# From the editors: Reflections on the nexus of complementarity between international business research and the policy practitioner community

**DOI:** 10.1057/s42214-021-00111-y

**Published:** 2021-04-27

**Authors:** Sarianna Lundan, Ari Van Assche

**Affiliations:** 1 Faculty of Business Studies and Economics, University of Bremen, Enrique-Schmidt-Strasse 1, Bremen, 28359 Germany; 2 HEC Montréal, 3000 chemin de la Côte-Sainte-Catherine, Montréal, H3T2A7 Québec Canada

## Introduction

International Organizations (IOs) such as UNCTAD, the World Bank and the OECD are tasked with promoting sustainable development in the global economy. This requires an in-depth understanding of the international trade and investment landscape, and in this respect, recent shifts in the patterns of globalization present a major challenge to IOs. They need to develop new knowledge that helps to identify the implications of these trends for the links between trade, foreign investment and development. What is driving the state of flux in the trade and investment landscape? Do the new global realities strengthen or weaken the abilities of countries to grow through trade and investment? What policies should countries adopt to harness the new global (dis)order?

The purpose of this special collection is to highlight the role that international business (IB) scholarship can play in addressing the needs of IOs for new knowledge that is relevant to global economic policymaking. We do this by first presenting a forward-looking perspective by James Zhan, Director of Investment and Enterprise at UNCTAD, on the challenges that lie ahead over the next decade. This is followed by three commentaries from senior scholars within the IB community, namely Peter Buckley, Lorraine Eden with Niraja Srinivasan, and Karl P. Sauvant, each having a different viewpoint on the drivers of change and key actors in the global economy.

In previous editorials, we have argued that if they make a ‘policy turn’ in their research, IB scholars are natural allies for IOs to develop evidence-based answers to these time-sensitive questions (Lundan, 2018; Van Assche, 2018). As a result of their dominant role in world trade, and their unique impact as foreign investors on both the the home and host countries, multinational enterprises (MNEs) drive many of the transformations in the global economy (Dunning & Lundan, 2008). Insights into the forces that propel MNEs to reconfigure their activities, strategies and structures are thus key building blocks for understanding the latest IB trends and their implications, and this is precisely the type of expertise that IB scholars have to offer (Buckley et al., 2017).

We start with an overview of the articles included in the special collection, using them to illustrate the various ways in which IB scholarship can form a nexus of complementarity with the analytical and advisory work performed by IOs. We then discuss the extra steps that IB scholars should take to make their research even more relevant for policy, emphasizing the need for more phenomenon-based and policy-oriented research.

## Papers in this special collection

In his lead article, Zhan (2021) lays out the significant implications that transforming global value chains (GVCs) can have for the global trade and investment landscape and MNEs’ modes of operation. He identifies five major forces that are currently driving GVCs to transform, which include a deepening of geopolitical tensions that is weakening the global governance system, the digitalization of supply chains, sustainability becoming a corporate imperative, firms being increasingly held accountable for their sustainability actions, and socio-economic trends that are globally increasing volatility, uncertainty, complexity and ambiguity (VUCA). He then presents ten broad ways in which these forces will reconfigure both GVCs and the global trade and investment landscape. Finally, he concludes with a list of policy-relevant questions that these trends raise, opening the door for IB scholars to contribute to the policy debates at UNCTAD and elsewhere.

The first commentary by Buckley (2021) focuses on the usefulness of IB theories to study the influence of the major forces identified by Zhan on the global trade and investment landscape. According to Buckley, IB theory lends itself to studying the effects of exogenous shocks such as geopolitical shifts and heightened VUCA on GVC structures by tracing how they influence MNEs’ internalization, location and governance decisions. At the same time, he cautions that deeper reflection is needed on the implications of partially endogenous forces such as digitalization and the sustainability imperative for GVCs.

While the adoption of digital technologies can induce the adaptive restructuring of MNEs’ production activities, the reverse is also possible. Indeed, since digitalization is a technology choice that MNEs make, a change in GVC structure (potentially driven by other major forces) can persuade an MNE to develop or adopt digital technologies (see also Ambos et al., 2021), introducing important interactions between major change forces. Similarly, MNEs’ mainstreaming of sustainability is not simply the result of exogenously driven stakeholder and governmental pressure, as MNEs have direct influence on civil society and policy at all levels of formulation and implementation, and these actions affect the type of pressures that these actors put on MNEs. Buckley thus calls for deeper theoretical and empirical analysis of both the national and global context in which Zhan’s major forces have emerged, as well as how these different forces interact with each other.

Srinivasan & Eden (2021) fully embrace Buckley’s call to study the interaction between the major forces identified by Zhan. In their commentary, the authors build on business insights to ask the question whether MNEs can adopt digital technology in such a way that it also helps them to embrace the sustainability imperative. Despite the dark sides of digitalization (Verbeke & Hutzschenreuter, 2020), MNEs can clearly use digital technologies for the good. The digitalization of supply chains can help MNEs to reduce waste, increase transparency, improve traceability, and enhance worker safety. Digital technology combined with smart meters and sensors also allows MNEs to measure the impact of their actions more accurately, thus giving them the opportunity to internalize some of the negative externalities that they generate.

In spite of this potential, Srinivasan and Eden point out that MNEs need important behavioral changes to turn these digital-based opportunities into a sustainability-supporting reality. First, MNEs need to revamp their corporate social responsibility (CSR) function to ensure that sustainability considerations are fully taken into account when digital technology is adopted. Second, MNEs would benefit from linking their CSR function to the United Nations’ sustainable development goals (SDG) so that the SDG mindset gets mainstreamed. The authors suggest that IOs can play a key role in pushing the SDG mindset among MNEs. Specifically, they propose that UN organizations such as UNCTAD could foster a new public-private partnership with a coalition of willing MNEs that identifies and monitors the commitments that MNEs make.

Sauvant’s (2021) commentary differs from the other two in that it takes a government instead of an MNE perspective. Furthermore, by anchoring his analysis in the international law and policy regime, Sauvant is particularly concerned with the institutions that regulate and shape MNE behavior. The central question he asks is what can governments do to increase the benefits they derive from FDI. How can they encourage more sustainable FDI? And what can they do to enhance the distribution of benefits associated with FDI? The author argues that a more pro-active “green” industrial policy towards FDI can go a long way in fulfilling these goals. Governments can make special efforts to facilitate FDI projects whose characteristics are most likely to advance countries’ sustainable development. They can do this unilaterally by prioritizing FDI in certain industries or by facilitating FDI by so-called “Recognized Sustainable Investors.” They can also do this multilaterally by including sustainable FDI in the current WTO negotiations for an Investment Facilitation Framework for Development (IFF4D). For these policies to be effective, however, detailed knowledge about the motivations and strategies of MNEs is needed.

Taken together, the special collection portrays three ways in which IB scholars can engage with IOs to develop policy-oriented trade and investment knowledge: by providing *IB theories* that can explain new trends (Buckley, 2021); by presenting *business insights* that can be used to develop new theories (Srinivasan & Eden, 2021); and by designing *evidence-based policies* that can harness new global realities (Sauvant, 2021). In the next section, we discuss the steps IB scholars could take to conduct more phenomenon-based and policy-relevant research.

## Increasing policy relevance

Both Buckley (2021) and Srinivasan and Eden (2021) take an actor-based view where the MNE both reacts to and acts upon other players in the economy. While Buckley (2021) emphasizes the partly endogenous nature of some of the ongoing changes in the global economy, Srinivasan and Eden (2021) examine the strategic choices available to MNEs in the context of profound technological and political change. Both perspectives are evolutionary in the sense that multinational firms react to changes in their environment and introduce new technologies and governance solutions to ensure continued viability (Lundan & Cantwell, 2020). Aside from extreme periods such as the COVID-19 pandemic when governments intervene in markets with a heavy hand, the co-evolutionary process between firms and other actors in the economy changes the configuration of GVCs in incremental steps. Over time, these adjustments add up to changes at the systemic level, and influence both governments and civil society as well as the MNEs’ customers and competitors. It is precisely this process of cumulative changes to the organization of production, and the development and introduction of new technologies, that IB scholarship has examined in considerable detail.

**Figure 1:**
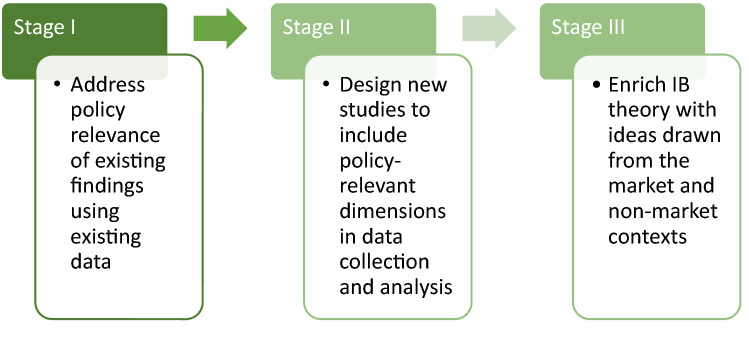
Increasing policy relevance in IB research.

However, much of the IB scholarship has approached these issues from the perspective of firms and highlighting their implications for management. Although these studies have not been designed to address policy issues, we believe that they can nonetheless have relevance for policy, as they reveal the decision-making processes and strategic alternatives available to MNEs. In terms of the development of more policy-oriented research, we call these Stage I studies (see Figure 1). It is our goal at JIBP to encourage IB scholars to engage more deeply with the potential policy relevance of their findings from Stage I studies, and to look for opportunities to develop new insights by linking their research to other areas of literature and to the work of policy professionals.

Stage II of this process involves studies where the research design includes questions and outcomes that have relevance not only for the MNE but also for policymakers. For such studies to be truly impactful, the views and concerns of policy practitioners should already be included at the design stage (Simsek et al., 2018). This requires IB scholars to embrace phenomenon-based research and build their research design around real-world phenomena that lie at the intersection of business and society (Doh, 2017). To facilitate this connection, we have actively sought to include policy professionals from IOs such as the OECD and think tanks such as the Brookings Institution to become engaged with the journal and to share their views on the big policy issues of today (see, e.g., the COVID-19 special series or the special issue on the SDGs). Additionally, Stage II research projects will certainly benefit from the more structured collaboration that is beginning to take place between AIB and UNCTAD that includes joint webinars, awards for young scholars to visit UNCTAD, and participation in the bi-annual World Investment Forum.

Over time, a successful ‘policy turn’ should then lead to Stage III studies, that enrich the conceptual and theoretical understanding within the field of IB. In terms of the nexus of collaboration with the policy community, these contributions are likely to have less direct relevance than Stage II studies. However, they are immensely important for securing the position of JIBP as an academic outlet that publishes the very best original research and conceptual contributions on IB policy that can become the career-defining pieces particularly for younger scholars.

## Conclusion

At the end of his article, Zhan (2021) presents a list of questions that the five major forces and the transformation of GVCs bring about. These questions have two features in common. First, they focus on “grand challenges” where innovative scholarship is needed to tackle problems that affect both MNEs and the societies in which they are embedded (Buckley et al., 2017). Second, they concentrate on the actions that governments can take to better harness the reconfigured GVCs.

We would like to make the case that IB research will be particularly well-placed to answer these questions if researchers proactively embraced grand challenges and policy questions in their research. We thus join the call for IB scholars to incorporate both phenomenon-based and policy-oriented research into their research designs (Doh, 2017). One way to measure the impact of academic research is by the visibility of the output, such as citations to individual articles. Another way to understand impact is to see it as being reflected in the research process that engages practitioners at different stages. The dual mission of JIBP is to provide a premier outlet for the academic output, as well as to offer a platform for linking the academic and policy professional communities through, e.g., invited commentaries and appearances in joint events and webinars.

We think that the piece by Zhan (2021) offers a compelling bird’s eye view on the totality of challenges that confront the global economy today. While remaining optimistic that there are solutions to these challenges, this special collection suggests that we need even more dialogue between the policy practitioners and the academic community in order for IB scholarship to play a more active role in the discussions that are re-shaping GVCs and the global economy as a whole.
